# Association of Low Molecular Weight Plasma Aminothiols with the Severity of Coronavirus Disease 2019

**DOI:** 10.1155/2021/9221693

**Published:** 2021-09-18

**Authors:** Evgeny Vladimirovich Kryukov, Alexander Vladimirovich Ivanov, Vladimir Olegovich Karpov, Valery Vasil'evich Alexandrin, Alexander Mikhaylovich Dygai, Maria Petrovna Kruglova, Gennady Ivanovich Kostiuchenko, Sergei Petrovich Kazakov, Aslan Amirkhanovich Kubatiev

**Affiliations:** ^1^Burdenko Main Military Clinical Hospital, Ministry of Defense, Gospitalnaya Sq., 3, 105229 Moscow, Russia; ^2^Institute of General Pathology and Pathophysiology, Baltiyskaya St., 8, 125315 Moscow, Russia; ^3^Federal State Autonomous Educational Institution of Higher Education I.M. Sechenov First Moscow State Medical University of the Ministry of Health of the Russian Federation (Sechenov University), Trubetskaya St., 8-2, 119991 Moscow, Russia; ^4^Regional Clinical Hospital, Lyapidevsky St., 1, 656024 Barnaul, Russia

## Abstract

**Objective:**

Aminothiols (glutathione (GSH), cysteinylglycine (CG)) may play an important role in the pathogenesis of coronavirus disease 2019 (COVID-19), but the possible association of these indicators with the severity of COVID-19 has not yet been investigated.

**Methods:**

The total content (*t*) and reduced forms (*r*) of aminothiols were determined in patients with COVID-19 (*n* = 59) on admission. Lung injury was characterized by computed tomography (CT) findings in accordance with the CT0-4 classification.

**Results:**

Low tGSH level was associated with the risk of severe COVID-19 (tGSH ≤ 1.5 *μ*M, mild vs. moderate/severe: risk ratio (RR) = 3.09, *p* = 0.007) and degree of lung damage (tGSH ≤ 1.8 *μ*M, CT < 2 vs. CT ≥ 2: RR = 2.14, *p* = 0.0094). The rGSH level showed a negative association with D-dimer levels (*ρ* = −0.599, *p* = 0.014). Low rCG level was also associated with the risk of lung damage (rCG ≤ 1.3 *μ*M, CT < 2 vs. CT ≥ 2: RR = 2.28, *p* = 0.001). Levels of rCG (*ρ* = −0.339, *p* = 0.012) and especially tCG (*ρ* = −0.551, *p* = 0.004) were negatively associated with platelet count. In addition, a significant relationship was found between the advanced oxidation protein product level and tGSH in patients with moderate or severe but not in patients with mild COVID-19.

**Conclusion:**

Thus, tGSH and rCG can be seen as potential markers for the risk of severe COVID-19. GSH appears to be an important factor to oxidative damage prevention as infection progresses. This suggests the potential clinical efficacy of correcting glutathione metabolism as an adjunct therapy for COVID-19.

## 1. Introduction

The treatment of coronavirus disease 2019 (COVID-19) caused by severe acute respiratory syndrome coronavirus-2 (SARS-CoV-2) has become a focus of medical research since 2020. Endothelial dysfunction plays a key role in the pathogenesis of this condition, and the disease develops as a result of the disruption of the surface protein angiotensin II-converting enzyme (ACE II). This triggers numerous pathways that dysregulate the homeostasis of vascular tone and permeability and leads to impaired lung function and in some cases to multiple organ failure [1].

Markers of COVID-19 severity and prognosis are being actively studied. Several studies have suggested that a precise disulfide-thiol balance is crucial for viral entry and fusion to the host cell and that oxidative stress generated from free radicals can affect this balance [2]. Low molecular weight aminothiols (LMWTs: cysteine (Cys), cysteinylglycine (CG), glutathione (GSH), and homocysteine (Hcy)) play an important role in biochemical processes involved in the key mechanisms of the body's response to COVID-19; therefore, they can also be considered potential biomarkers of the severity of COVID-19. GSH is the main intracellular antioxidant, and glutathionylation of proteins is one of the important mechanisms of posttranslational regulation of their function [3]. Low GSH levels are associated with a predisposition to respiratory tract infections and cardiometabolic disorders [4, 5]. GSH and Cys were found as independent factors of atherothrombotic events [6]. In plasma, GSH is hydrolyzed to CG. For the synthesis of GSH, Cys is required, which can be formed as a result of hydrolytic cleavage of proteins, acquired from the extracellular environment or synthesized from Hcy by the so-called transsulfuration pathway. Hcy is formed from methionine via the intermediates S-adenosylmethionine and S-adenosylhomocysteine. Due to the fact that all transmethylation reactions use S-adenosylmethionine as a methyl group donor, Hcy has an impact on many vital processes, including the regulation of gene expression for cytokines, inflammatory proteins, and proliferation of viral particles.

Elevated Hcy levels (hyperhomocysteinemia (HHcy)) may be an important factor affecting the negative course of COVID-19 since it affects key pathophysiological mechanisms: oxidative stress (OS), endothelial dysfunction, thrombosis, and activation of type 1 receptors to angiotensin II (AT1R) [7–9]. In contrast, OS, which is characteristic of systemic inflammatory diseases, negatively affects the basic pathways of Hcy utilization (methionine synthase and betaine homocysteine methyltransferase activity, which can lead to an increase in Hcy plasma levels [10–12]. In blood plasma, LMWTs exist mainly in the oxidized form, and only a small proportion is in the reduced (*r*) form [13]. The sum of these forms is the total (*t*) content of LMWTs.

Despite the high interest in COVID-19, its effects on the LMWT system have not yet been reported. Several studies have suggested that high Hcy and especially a GSH deficit are risk factors for the severity of COVID-19 or its complications [2, 14–20]. One study demonstrated that a high tHcy can predict severe pneumonia on chest CT in COVID-19 patients [21]. The same study found that a tHcy level exceeding 15.4 *μ*M increases the probability of COVID-19 progression to extremely severe forms of severe acute respiratory syndrome by 3.2–3.5-fold.

In this study, we investigated the plasma levels of the *r*- and *t*-forms of Hcy, Cys, CG, and GSH in COVID-19 patients, in order to identify their associations with traditionally used laboratory parameters and advanced oxidation protein products (AOPP, a marker of oxidative stress (OS)) and to identify the possible impact of these LMWTs on COVID-19 severity and level of lung injury.

## 2. Methods

### 2.1. Patients

**Figure 1:**
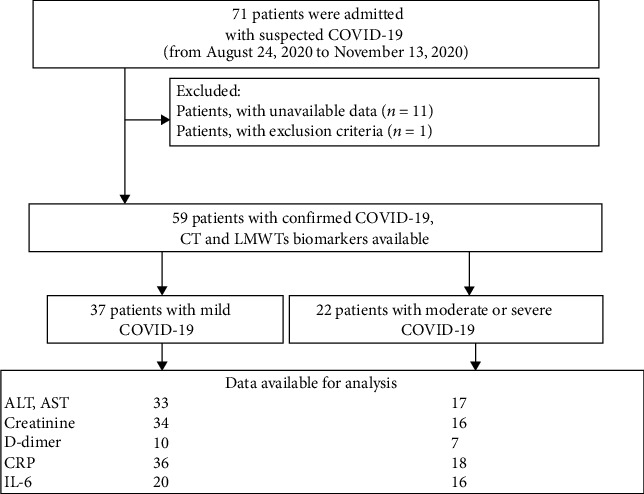
Patient flow diagram.

This study included 59 COVID-19 patients who were admitted to the pulmonary department of the Burdenko Main Military Clinical Hospital from August 24 to November 13, 2020. The study was approved by the local institutional ethics committee. Informed written consent was obtained from each patient. A graphical scheme of study design is presented in Figure 1.

The patients were diagnosed according to the World Health Organization interim guidance for COVID-19. The main inclusion criterion was a confirmed primary SARS-CoV-2 infection. Exclusion criteria included the following: exacerbations of cardiovascular disease, HIV infection, hepatitis B and C, terminal cancer, and decompensated renal failure. All patients included in the study were discharged with recovery from infection and improvement in their general condition.

Chest CT scans were performed on the 48 h of patients' admission using the Optima CT660 tomograph (GE Healthcare, USA), from the level of the thoracic entrance to the level of the diaphragm, and completed at the end of inspiration. The scanning parameters were as follows: tube voltage 120 kV, tube current 114~ 350 mA, and layer thickness 5 mm. At the end of scanning, a thin layer image with a layer thickness of 2.5 mm is automatically reconstructed and recorded as DICOM image data. The reconstruction algorithm used is with a field of view of 360 mm × 360 mm and a matrix of 512 × 512. Image browsing and multiplane reconstruction were performed using GE AW VolumeShare software v.4.6; images of the lungs (window width 1500, window level 500) and the mediastinum (window width 350, window level 35-40) were also observed using the same software. Image analysis was performed based on the standard protocol as described elsewere [22]. The degree of lung damage then was assessed using the following scoring system based on percentage of lobar involvement: <5% (CT0), 5-25% (CT1), 26-49% (CT2), 50-75% (CT3), and >75% (CT4) [23]. Based on the data of an objective study, respiratory function, and blood oxygen saturation, patients were categorized as having mild, moderate, or severe COVID-19 using previously described criteria above [24].

### 2.2. Laboratory Procedures

Venous blood samples were collected upon admission in tubes containing sodium citrate (0.105 M) and centrifuged at 3000*g* for 15 minutes. Then, plasma (1450 *μ*l) was mixed with 3 M acetic acid (50 *μ*l) and samples were frozen at -80°C and stored until LMWTs determination.

All patients were confirmed by viral detections using the SARS-CoV-2 nucleic acid detection kit “AmpliPrime® SARS-CoV-2 DUO” (Next Bio, Russia) and PCR analyzer RotorGene Q (Qiagen, Germany). Hematology analyzer MD-7600 (Meredith Diagnostics, United Kingdom), automatic biochemistry analyzer Ellipse (Analyzer Medical System, Italy), Biosen C line (EKF Diagnostics, Germany), express immunochemiluminescent analyzer PATHFAST (Mitsubishi Chemical Medience Corporation, Japan), and erythrocyte sedimentation rate analyzer ESR 3000 (SFRI, France) were used for routine blood analysis.

LMWTs were determined by liquid chromatography as described early with some modifications [25]. An UPLC ACQUITY system (Waters, Milford, MA) with a PDA*λ* UV-detector (*λ* = 330 nm) and FTN Sample manager was used.

For total LMWT determination, we mixed 5 *μ*l of 1 mM penicillamine (internal standard), 5 *μ*l of 0.2 M dithiothreitol, and 10 *μ*l of 0.4 M Na-phosphate buffer pH 8.0, containing 50 mM ethylenediaminetetraacetic acid disodium salt, with 50 *μ*l of blood plasma. These mixtures were incubated for 30 minutes at 37°C, and 200 *μ*l of 5,5′-dithiobis(2-nitrobenzoic) acid (DTNB) in acetonitrile was added. Probes were mixed intensively and centrifuged for 5 minutes at 15,000*g*. We then added 10 *μ*l of 1 M HCl and 200 *μ*l CHCl_3_ to each supernatant. Probes were mixed intensively and centrifuged for 1 min at 4,000*g*. The upper phase was diluted 3 times and injected (10 *μ*l) in chromatograph. A Zorbax Eclipse Plus C18 Rapid Resolution HD 100 × 2 mm and 1.8 *μ*m column (Agilent, Santa Clara, CA) was used for quantitation of total LMWTs. The flow rate was 0.15 ml/min, and *t* = 35°C. Mobile phases were 0.15 M NH_4_ acetate with 0.075% (*v*/*v*) formic acid and acetonitrile. Chromatography was performed using a linear acetonitrile gradient (3%–13%) for 4.5 minutes. The column was regenerated with 50% acetonitrile for 0.5 minute and equilibrated with 3% acetonitrile for 6.5 minutes.

For reduced LMWT determination, 200 *μ*l of plasma was mixed with 400 *μ*l 2.5 mM DTNB in acetonitrile and 12 *μ*l of 1.5 M NaOH. Afret 5 sec 20 *μ*l of 0.1 M iodoacetamide was added. After 10 minutes, incubation samples were centrifuged for 5 minutes at 15,000*g*. Then, the supernatant (200 *μ*l) was mixed with 50 *μ*l of internal standard (10 *μ*M penicillamine+200 *μ*M DTNB) and 1200 *μ*l water, and the mixture was passed through a diethylaminoethyl cellulose column (40 mg). The column was flushed with 2 ml water, and analytes were eluted with 400 *μ*l 0.2 M HCl with 0.2 M NaCl. A Zorbax Eclipse Plus C18 Rapid Resolution HD 150 × 3 mm and 1.8 *μ*m column (Agilent, Santa Clara, CA) was used for quantitation of reduced LMWTs. The flow rate was 0.4 ml/min, and *t* = 40°C. Mobile phases were 0.05 M NH4 acetate with 0.15% (*v*/*v*) formic acid and acetonitrile. Chromatography was performed using a linear acetonitrile gradient (4%–11.5%) for 3 minutes. The column was regenerated with 50% acetonitrile for 0.5 minute and equilibrated with 4% acetonitrile for 4.2 minutes.

Advanced oxidation protein product (AOPP) level in plasma was determined by the Witko-Sarsat method [26] with little modifications. After centrifugation (1 min, 1000*g*), the plasma sample (80 *μ*l) was mixed with PBS+0.05% Nonidet P40 (320 *μ*l), KI (1.16 M, 20 *μ*l), and acetic acid (40 *μ*l). Optical density (OD) was immediately measured at 340 nm against a blank sample (PBS with KI and acetic acid) in a 1 cm path cuvette. Chloramine B (0–62.5 *μ*M) in PBS was used as calibration standards. Its absorbance was linear within this range (OD = 0.0156·C^chloramine^+0.0059, *R*^2^ = 0.999).

### 2.3. Statistical Analysis

Data collection and primary processing (identification and integration of the chromatographic peaks) were performed in MassLynx v4.1 (Waters, USA). Statistical data analysis was performed using SPSS Statistics v. 22 (IBM, USA). Data on age, clinical findings, biochemical tests, and LMWT levels were expressed as medians (1st; 3rd quartile). Differences in the levels of these parameters between the patient groups were determined using the Mann–Whitney *U* tests. Spearman's correlation coefficient (*ρ*) was used to measure the degree of association between two variables. Binomial indicators (bivariable analysis) were compared by calculating the relative risk (RR) and odds ratio (OR). A *p* value < 0.05 was considered to indicate a significant difference.

## 3. Results

The general characteristics of patients are presented in Table 1. Most patients (46 of 59) were men. We found no statistically significant difference in sex distribution in the mild and moderate/severe groups. The median patient age was 61 (range, 20–88) years. No smokers or regular users of alcohol or drugs were identified. Most of the admitted patients had mild COVID-19 (68%) and no more than 50% lung damage (75%). Only three (5%) patients had severe COVID-19, and two of them had a degree of lung damage that corresponded to CT4. Therefore, the groups with moderate and severe COVID-19 and CT3 and CT4 were subsequently merged. On admission, two patients underwent resuscitation/intensive therapy. A significant proportion of patients were previously diagnosed with arterial hypertension (24 out of 59, 41%) and atherosclerosis (17 out of 59, 29%). Sixteen (27%) patients were diagnosed with HHcy (tHcy > 10 *μ*M), mostly mild (<15 *μ*M). Only 6 patients had a tHcy level > 15 *μ*M.

Spearman's rank correlation revealed a number of associations between clinical laboratory parameters and LMWTs in all the patients. The tCys level had a negative impact on hemoglobin (HGB) level (*ρ* = −0.330, *p* = 0.0093, *n* = 59). The tCG level was positively associated with hematocrit (HCT: *ρ* = 0.395, *p* = 0.0128, *n* = 39) and had a rather significant negative effect on platelet count (PLT: *ρ* = −0.551, *p* = 0.00041, *n* = 37). Level of rCG was also negatively associated with PLT (*ρ* = −0.339, *p* = 0.0121, *n* = 54). A negative relationship was found between tGSH levels and mean erythrocyte volume (MCV: *ρ* = −0.425, *p* = 0.0011, *n* = 56) and mean erythrocyte hemoglobin (MCH: *ρ* = −0.449, *p* = 0.00051, *n* = 56). Interestingly, a fairly close negative association was observed between D-dimer and rGSH levels (*ρ* = −0.599, *p* = 0.0142, *n* = 16), although D-dimer level determination was performed in a limited number of patients (*n* = 16).

Since there were only 3 patients with severe infection in the cohort, the patients were stratified into two groups based on disease severity (mild and moderate+severe). No significant differences in age and sex were found in these groups. As shown in Table 2, patients with moderate or severe disease were characterized by increased incidence of severe lung injury (CT3-4), hemoconcentration (increased HCT), increase in the leukocyte index, and decrease in tGSH levels.

When comparing patients with different degrees of lung damage, significant differences were observed in a number of indicators (Table 3). The erythrocyte sedimentation rate (ESR) and C-reactive protein (CRP) level significantly increased in the CT0-CT4 series. Among LMWTs, it can be noted that tGSH and rCG levels were lower in CT2-4 patients than in CT0-1 patients.

The impact of LMWTs (tGSH, rCG) as risk factors for the severity of COVID-19 and lung injury is presented in Tables 4 and 5. As shown in Table 4, tGSH levels ≤ 1.5 *μΜ* corresponded to 3-fold higher risk of moderate/severe COVID-19. Approximately 80% of patients with moderate/severe COVID-19 had tGSH levels ≤ 1.5 *μ*M. Low levels of rCG (≤1.3 *μ*M) and tGSH (≤1.8 *μ*M) were also associated with at least the double risk of moderate-to-severe lung damage (Table 5). Most of the CT2-4 patients (83%) had low tGSH and rCG levels. The RR of high lung damage (CT3-4) in patients with low tGSH and rCG levels was 3-fold higher than that in patients with tGSH > 1.8 *μ*M or rCG > 1.3 *μ*M.

**Figure 2:**
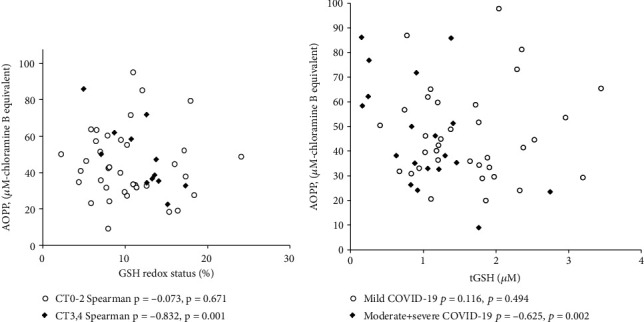
Association of AOPP with GSH redox status (a) and tGSH level (b).

We did not find any significant differences in AOPP levels when comparing patients with different severity or degrees of lung injury. We also did not find any significant association of this indicator with *r*- and *t*-forms of LMWTs and their *r*/*t* ratio in the entire cohort of patients. However, it was found that in patients with severe lung injury (CT3,4), there is a strong negative association between AOPP and *r*/*t* of the GSH ratio (i.e., its redox status), which is absent in patients with moderate lung injury CT0-2 (Figure 2(a)). In addition, a negative association of AOPP with tGSH was observed in the group of patients with moderate or severe infection, which was not observed in patients with mild COVID-19 (Figure 2(b)).

## 4. Discussion

In a previous large study of 273 patients with COVID-19, negative progression in the lungs on CT was associated with the level of tHcy and the role of HHcy as a factor for progression to severe COVID-19 [21]. In contrast, in our work, there was no significant effect of tHcy and rHcy on the severity of infection, and there was no significant association of these parameters with the results of a clinical blood test. However, it is worth noting that the frequency of HHcy in patients with moderate/severe COVID-19 at admission was twice as high as that in patients with mild COVID-19 (42 vs. 20%, *p* = 0.075), but the sample size was not large enough for this difference to be significant. In addition, there were no patients in our sample whose disease progressed and who did not recover.

OS plays an important role in the pathogenesis of atherosclerosis and inflammatory lung diseases. Although the details of GSH involvement in these processes are not yet fully understood, the importance of the protective function of this aminothiol is emphasized both by its direct antioxidant activity and the key role of GSH-dependent enzymes that carry out (de)glutathionylation of proteins and the GSH hydrolysis. The role of glutathione S-transferase (GST) P1, glutathione transferase omega 1 (GSTO1-1), *γ*-glutamyl transpeptidase (GGT), and glutaredoxin in the activation of endothelial cells, smooth muscle cells, and macrophages is being actively studied [27–30]. The deficiency of the GSH redox cycle (GSH-peroxidase and GST) enzymes observed in the atherosclerotic plaques area contributes to the creation of a prooxidant environment within the vascular wall [31]. The protective role of GST P1 was also demonstrated in a model of endotoxinemia, where it was shown that activation of this enzyme causes inhibition of MAPKs and NF*κ*B, which leads to suppression of the expression of proinflammatory factors TNF*α*, IL-1*β*, MCP-1, and overproduction of NO [32].

In addition, it was found that the effect of oxidized low-density lipoproteins (LDL) in macrophages leads to an increase in the level of glutathionylation of their proteins and promotes cell death [33]. It was revealed that GSTO1-1 plays an important role in the activation of macrophages by deglutathionylation of proteins such as caspase-1, STAT3, and hypoxia-inducible factor 1*α* [28], and GSH protects macrophages from oxidized LDL-induced cell injury [31]. On the other hand, the ApoB100 protein, which is part of LDL, is itself a target of glutathionylation, but the pathological significance of this modification has not yet been adequately studied. Glutathionylation of proteins is a mechanism actively involved in the formation of atherosclerotic plaque and ED in general, including oxidation of LDL, modulates cell response to OS in key events of plaque initiation (monocyte recruitment and differentiation), and progression (macrophage activation and death) [31, 33]. This is confirmed by a clinical study in which the positive correlation between atherosclerosis progression and the level of protein glutathionylation was found [34]. Among the numerous targets, one can distinguish Ca^+2^ ATPase, whose glutathionylation is a cGMP-independent mechanism of vasodilation, impaired in atherosclerosis [35]; the regulatory protein Ras is activated upon glutathionylation under the action of various atherogenic stimuli (angiotensin II, peroxynitrile, and oxidized LDL) and triggers the activation of Akt and ERK [33]. Interestingly, the Ras mutation, which prevents its glutathionylation, blocks the development of OS mediated by angiotensin II [36]. Glutathionylation of glutaredoxin-1 is also likely to be involved in the regulation of the Akt-dependent signaling pathway and is important for maintaining the physiological level of vascular permeability [33, 37]. In addition, the disturbance of laminar flow, observed in the areas most susceptible to atherosclerotic changes, negatively affects the activity of glutaredoxin-1 [30].

Our results showed that low tGSH levels could be considered a marker for the risk of developing severe COVID-19, severity of lung damage, and course of COVID-19. Although there is very little data on the role of GSH during COVID-19, there are some reasons that this metabolite may play an important role in viral replication and resistance to infection [20]. It is known that loss of GSH affects the Na^+^ H^+^ membrane antiport with the decrease in intracellular pH, which facilitates both virus endocytosis and its replication [5]. It has been concluded from previously published literature that various risk factors associated with high mortality rates of COVID-19 are usually associated with low baseline GSH levels or impaired GSH metabolism; thus, GSH depletion may play a central role in COVID-19 mortality and pathophysiology [19]. The role of GSH in the protection of DNA from peroxynitrile-mediated damage, which is characteristic of acute inflammatory reactions, has been shown early [16].

AOPP was proposed in 1996 by Witko-Sarsat et al. as a surrogate marker of OS [26]. AOPP are formed mainly due to the oxidation of tyrosine residues and SH-groups of plasma proteins by hypochlorous acid (HClO), which, in turn, is formed by the reaction of H_2_O_2_ with Cl^−^, catalyzed by myeloperoxidase. The appearance of a close negative association of this indicator with the level of plasma tGSH and GSH redox status in patients with high level of lung injury indicates that GSH is becoming a really important determinant that prevents oxidative damage to proteins. An indirect confirmation of the results obtained is the close correlation between the level of rGSH in blood and the level of SH-groups of proteins in critically ill adult patients hospitalized for severe COVID-19 [38].

With COVID-19, there is a shift in the balance between angiotensin II, which has a prooxidant effect, and angiotensin 1-7, the ACEII-mediated product of angiotensin II cleavage, which inhibits reactive oxygen species (ROS) generation. Overall, the formation of OS is favored. This can enhance the invasion of SARS-CoV-2 due to the oxidation of cysteine residues in ACE-II and the virus's spike glycoproteins [39]. Angiotensin II-mediated activation of NADPH oxidase can have a profound effect on GSH homeostasis, since the restoration of GSH by GSH reductase requires NADPH. It is interesting to note that in addition to these, in principle, nonspecific mechanisms, two more mechanisms related to GSH metabolism, specific for COVID-19, have been suggested [17]. First, it was hypothesized that the major protease SARS-CoV-2 has the ability to break down GSH peroxidase, the main enzyme that GSH uses to detoxify cells from ROS. Second, it has been suggested that this major protease can cleave glutamate-cysteine ligase, which is necessary for the synthesis of GSH. In addition, SARS-CoV-2-mediated activation of tumor growth factor (TGF-*β*) suppresses the expression of glutamate-cysteine ligase [40]. Finally, it is known that many viruses are capable of activating mechanisms aimed at reducing the synthesis and redox status of GSH by increasing the expression of NADPH oxidases, NF-*κ*B, and inhibition of NRF2 expression [5].

Low GSH levels inhibit T lymphocyte proliferation and subsequently disrupt the immune response [41, 42]. GSH depletion is necessary for apoptosis to be triggered in lymphocytes, regardless of the presence of ROS [43]. GSH may also contribute to the increased risk of severe COVID-19 with age, since in the elderly, there is a decrease in the GSH level in erythrocytes, lymphocytes, and plasma [44–46].

Although the results of studies on the role of vitamin D deficiency in the severity of COVID-19 are ambiguous [47, 48], it has been shown that SARS-CoV-2 infection can inhibit the activity of the vitamin D receptor [49]. GSH deficiency can alter genes that work together to synthesize vitamin D, vitamin D-binding proteins, and receptors, but supplementation with L-cystine, a precursor for GSH, increases the levels of vitamin D and its binding proteins [20, 50].

The importance of the protective role of GSH in the development of SARS is confirmed by a number of works that show the effectiveness of the use of the GSH precursor N-acetylcysteine for the prevention of this complication in patients at high risk [51, 52]. Experimental work has also shown that GSH/N-acetylcysteine has antiviral activity toward a wide range of viruses [19, 20].

Numerous studies show that the fibrin degradation fragment, D-dimer, is a useful clinical indicator of thromboembolism, predictor of mortality, and marker for the progression of COVID-19 [20, 53, 54]. In this regard, it is interesting to identify a rather close negative association of rGSH with the D-dimer level, which may indicate a significant effect of GSH in the regulation of blood coagulation activity in COVID-19.

To date, data on the possibility of using GSH as a diagnostic marker or therapeutic target in COVID-19 are scarce. The first study that proposed a negative role of GSH deficiency in COVID-19 included a sample of only four patients, two of whom had severe and moderate-to-severe disease [18]. It was reported that these patients had a decreased plasma GSH/ROS ratios, but the method used to determine these values was not described. In patients in the intensive care unit (ICU), the level of rGSH in whole blood was reduced, and the GPX activity, on the contrary, was increased compared with reference levels [38]. So far, there are several reports of the successful use of N-acetylcysteine or GSH in patients with COVID-19 [55, 56].

In addition, of interest is the negative association of tGSH level with MCV and MCH in COVID-19 patients. In the literature, we did not find data on whether such an association exists in healthy individuals; therefore, we cannot argue that this relationship is a characteristic feature of COVID-19. However, it was previously shown that MCVs were significantly higher in COVID-19 nonsurvivors than in survivors, which indicates the importance of GSH as a protective factor [57].

The present study also found that patients with lung damage > 25% (CT2-4) had low plasma rCG levels. CG is a product of plasma GSH cleavage by the surface protein GGT. Decreases in rCG levels may be due to a shift in the redox status of LMWTs, a decrease in plasma rGSH (or tGSH) levels, a decrease in GGT activity, or an increase in dipeptidase levels. According to our data, nothing is currently known about changes in the activity of GGT and dipeptidase in COVID-19. Some clinical studies have revealed a positive association of GGT with the arterial hypertension, risk for cardiovascular diseases, and mortality [29]. Apparently, an increase in GGT activity is the body's response to OS [58], which is confirmed by the close relationship between the GGT level and CRP [59]. The level of Cys in the plasma and thus the synthesis of GSH largely depend on the activity of GGT and dipeptidase, since neither GSH itself nor CG is transported into cells. However, CG also has prooxidant properties, since, being a strong reducing agent, it is able to reduce Fe+3 to Fe+2. Oxidation of Fe+2, in turn, causes the appearance of ROS (superoxide anion, H_2_O_2_). It is with this mechanism that the participation of CG and GGT in atherosclerosis is associated [29]. It is also difficult to explain the negative association of rCG and tCG levels with PLT, especially in the latter case. It can be assumed that the activation of hemostasis, leading to a decrease in the PLT level, is accompanied by the release of GSH from platelets, which is rapidly metabolized to CG. Thus, the question of the association of CG with platelet functions requires additional research.

## 5. Conclusions

In the present work, it was shown that the levels of tGSH and rCG can be considered potential risk markers for the severity of COVID-19 and lung damage upon admission. GSH appears to be an important factor to oxidative damage prevention as infection progresses. Further, the association of GSH and CG with hematological parameters and D-dimer levels indicates the potential clinical efficacy of correcting GSH metabolism as an adjunct therapy for COVID-19. Since a decrease in GSH levels is characteristic of aging and comorbidities (diabetes mellitus, obesity, and hypertension), which can have a major impact on the development of COVID-19 severity [5], targeted studies of aminothiols among such patient groups are of interest.

## Figures and Tables

**Table 1:** General characteristics of patients^∗^.

*N*	59
Age, y	61 [51; 67.5]
Sex, man (%)	46 (78%)
Condition upon admission	
Mild	40 (68%)
Moderate	16 (27%)
Severe	3 (5%)
Severity of the infection:	
Mild	37 (63%)
Moderate	19 (32%)
Severe	3 (5%)
Arterial hypertension (%)	24 (41%)
Diabetes mellitus (%)	7 (12%)
Atherosclerosis (%)	17 (29%)
Lung CT level:	
0	2
1	24
2	17
3	13
4	2
HHcy: tHcy > 10 *μ*M (%)	16 (27%)
HGB (g/l)	144 [126; 154]
HCT (%)	42 [36.9; 44.5]
RBC (10^12^/l)	4.97 [4.16; 5.20]
MCV (fl)	86 [83; 89]
MCH (pg)	30.0 [28.6; 30.7]
MCHC (g/dl)	346.5 [335.0; 358.5]
PLT (10^9^/l)	260 [204; 312]
WBC (10^9^/l)	5.8 [3.9; 8.8]
LI	3.2 [1.9; 4.9]
ESR (mm/h)	46 [21; 76]
Glucose (mM)	5.55 [4.68; 7.03]
Creatinine (*μ*M)	93 [84; 112]
D-dimer (mg/l)	0.83 [0.48; 1.53]
CRB (mg/l)	23.5 [3.7; 55.7]
IL-6 (ng/l)	7.4 [2.9; 22.9]
LMWTs	
tCys (*μ*M)	227 [205; 249]
tCG (*μ*M)	13.6 [11.0; 16.3]
tGSH (*μ*M)	1.24 [0.92; 1.94]
tHcy (*μ*M)	7.9 [6.2; 10.4]
rCys (*μ*M)	14.8 [9.5; 16.9]
rCG (*μ*M)	1.43 [1.18; 1.90]
rGSH (nM)	163 [90; 223]
rHcy (nM)	93 [74; 127]
AOPP (*μ*M chloramine B equivalents)	41.8 [31.7; 60.7]

^∗^Data are presented as median [Q1; Q3].

**Table 2:** Comparative characteristics of patients with different severity of the course of coronavirus infection^∗^.

	Mild	Moderate+severe	*p*
*N*	37	22	
Age, y	63 [53; 68]	57.5 [50.5; 64.0]	0.373
Sex, man (%)	27	19	0.308
HHcy (tHcy > 10 *μ*M)	8	8	0.219
Arterial hypertension (%)	14 (38%)	10 (45%)	0.562
Diabetes mellitus (%)	3 (8%)	4 (18%)	0.246
Atherosclerosis (%)	11 (30%)	6 (27%)	0.84
Lung CT:			
0,1	19	7	0.144
2	12	6	0.67
3,4	6	9	0.045
HCT (%)	41 [33; 44]	43 [42; 45]	0.006
HGB (g/l)	138 [122; 152]	149 [141; 154]	0.038
LI	2.6 [1.6; 3.3]	4.1 [2.6; 5.7]	0.011
tGSH (*μ*M)	1.72 [1.10; 2.29]	0.99 [0.68; 1.40]	0.008

^∗^Data are presented as median [Q1; Q3].

**Table 3:** Comparative characteristics of patients with different degrees of lung damage.

	CT0, 1	CT2	CT3,4
*N*	26	18	15
Age, y	64.5 [51.3; 71.8]	60.5 [53.0; 66.5]	57.0 [49.5; 63.5]
Sex, man (%)	18 (69%)	13 (72%)	15 (100%)^‡^
HHcy: tHcy > 10 *μ*M (%)	4 (15%)	6 (33%)	6 (40%)
Arterial hypertension (%)	13 (50%)	6 (33%)	5 (33%)
Diabetes mellitus (%)	3 (12%)	2 (11%)	2 (13%)
Atherosclerosis (%)	9 (35%)	3 (17%)	5 (33%)
HGB (g/l)	140 [128; 161]	130 [120; 161]	147 [142; 149]
HCT (%)	42 [37; 45]	41 [36; 46]	42 [41; 43]
RBC (10^12^/l)	5.0 [4.3; 5.2]	5.0 [3.8; 5.3]	4.9 [4.5; 5.1]
PLT (10^9^/l)	261 [216; 326]	237 [169; 286]	271 [214; 317]
WBC (10^9^/l)	6.14 [4.68; 8.88]	5.2 [3.6; 5.9]	7.7 [4.1; 10.9]
ESR (mm/h)	34 [19; 52]	46 [27; 72]	82 [76; 86] ^‡,£,^^∗^^,^^∗∗^
D-dimer (mg/l)	0.93 [0.51; 1.53]	0.71 [0.46; 0.82]	1.23 [0.83; 1.53]
CRB (mg/l)	7.4 [1.8; 24.0]	38.9 [6.6; 56.0]^‡^	61.1 [32.0; 117]^‡,^^∗^^,^^∗∗^
IL-6 (ng/l)	4.85 [3.00; 18.5]	6.40 [3.48; 26.0]	13.97 [5.34; 50.6]
tGSH (*μ*M)	1.81 [1.04; 2.34]	1.15 [0.85; 1.76]	1.22 [0.76; 1.42]^∗^
rCG (*μ*M)	1.59 [1.31; 1.98]	1.30 [1.12; 1.78]	1.29 [1.08; 1.81]^∗^
AOPP (*μ*M chloramine B equivalents)	39.7 [29.5; 53.2]	42.0 [30.0; 61.1]	47.1 [34.3; 71.8]

^∗^CT0, 1 vs. CT2-4, ^∗∗^CT0-2 vs. CT3,4, ^‡^*p* < 0.05 compared with the “CT0,1” group. ^£^*p* < 0.05 compared with the “CT2” group.

**Table 4:** Effect of rCG and tGSH levels on the severity of lung injury.

rCG (*μ*M)	*N*_total_	*N* ^CT2-4^	*w* (%)	RR	*p*	OR	95% CI
≤1.3	23	18	78	2.28	0.001	6.9	2.05-23.2
>1.3	35	12	34				
tGSH (*μ*M)							
≤1.8	40	27	68	2.14	0.0094	4.5	1.39-14.5
>1.8	19	6	32				
	*N*_total_	*N* ^CT3-4^					
tGSH ≤ 1.8 and rCG ≤ 1.3 *μ*M	18	8	44	3.04	0.0132	4.67	1.3-16.6
tGSH>1.8 or rCG> 1.3 *μ*M	41	6	15				

**Table 5:** Influence of tGSH level on the severity of the course of coronavirus infection.

tGSH (*μ*M)	*N*_total_	*N* ^∗^	w (%)	RR	*p*	OR	95% CI
≤1.5	35	18	51	3.09	0.0068	5.29	1.50-18.7
>1.5	24	4	17				

^∗^Moderate+severe COVID-19.

## Data Availability

Data are available on request.

## Abbreviations

ACE II: Angiotensin II-converting enzyme
AOPP: Advanced oxidation protein products
AT1R: Type 1 receptors to angiotensin II
COVID-19: Coronavirus disease 2019
CT: Computed tomography
Cys: Cysteine
CG: Cysteinylglycine
CRP: C-reactive protein
DTNB: 5,5′-Dithiobis(2-nitrobenzoic) acid
ICU: Intensive care unit
GGT: *γ*-Glutamyl transpeptidase
GGT: *γ*-Glutamyl transpeptidase
GSH: Glutathione
GST: Glutathione S-transferase
GSTO1-1: Glutathione transferase omega 1
HCT: Hematocrit
HGB: Hemoglobin
Hcy: Homocysteine
HHcy: Hyperhomocysteinemia
LDL: Low-density lipoproteins
LMWTs: Low molecular weight aminothiols
MCH: Mean erythrocyte hemoglobin
MCV: Mean erythrocyte volume
OS: Oxidative stress
PLT: Platelet count
ROS: Reactive oxygen species
SARS-CoV-2: Acute respiratory syndrome coronavirus-2
TGF-*β*: Tumor growth factor beta.

## References

[1] Jin Y., Ji W., Yang H., Chen S., Zhang W., Duan G. (2020). Endothelial activation and dysfunction in COVID-19: from basic mechanisms to potential therapeutic approaches. *Signal Transduction and Targeted Therapy*.

[2] Suhail S., Zajac J., Fossum C. (2020). Role of oxidative stress on SARS-CoV (SARS) and SARS-CoV-2 (COVID-19) infection: a review. *The Protein Journal*.

[3] Musaogullari A., Chai Y. C. (2020). Redox regulation by protein S-glutathionylation: from molecular mechanisms to implications in health and disease. *International Journal of Molecular Sciences*.

[4] Ghezzi P. (2011). Role of glutathione in immunity and inflammation in the lung. *International Journal of General Medicine*.

[5] Rochette L., Ghibu S. (2021). Mechanics insights of alpha-lipoic acid against cardiovascular diseases during COVID-19 infection. *International Journal of Molecular Sciences*.

[6] Focks J. J., van Schaik A., Clappers N. (2013). Assessment of plasma aminothiol levels and the association with recurrent atherothrombotic events in patients hospitalized for an acute coronary syndrome: a prospective study. *Clinical Chemistry and Laboratory Medicine*.

[7] Škovierová H., Vidomanová E., Mahmood S. (2016). The molecular and cellular effect of homocysteine metabolism imbalance on human health. *International Journal of Molecular Sciences*.

[8] Schalinske K. L., Smazal A. L. (2012). Homocysteine imbalance: a pathological metabolic marker. *Advances in Nutrition*.

[9] Li T., Yu B., Liu Z. (2018). Homocysteine directly interacts and activates the angiotensin II type I receptor to aggravate vascular injury. *Nature Communications*.

[10] McCaddon A., Regland B., Hudson P., Davies G. (2002). Functional vitamin B(12) deficiency and Alzheimer disease. *Neurology*.

[11] Olteanu H., Banerjee R. (2001). Human methionine synthase reductase, a soluble P-450 reductase-like dual flavoprotein, is sufficient for NADPH-dependent methionine synthase activation. *The Journal of Biological Chemistry*.

[12] Miller C. M., Szegedi S. S., Garrow T. A. (2005). Conformation-dependent inactivation of human betaine-homocysteine S-methyltransferase by hydrogen peroxide in vitro. *The Biochemical Journal*.

[13] Ueland P. M., Mansoor M. A., Guttormsen A. B. (1996). Reduced, oxidized and protein-bound forms of homocysteine and other aminothiols in plasma comprise the redox thiol status--a possible element of the extracellular antioxidant defense system. *The Journal of Nutrition*.

[14] Ponti G., Ruini C., Tomasi A. (2020). Homocysteine as a potential predictor of cardiovascular risk in patients with COVID-19. *Medical Hypotheses*.

[15] Abu-Farha M., Al-Sabah S., Hammad M. M. (2020). Prognostic genetic markers for thrombosis in COVID-19 patients: a focused analysis on D-Dimer, Homocysteine and Thromboembolism. *Frontiers in Pharmacology*.

[16] Ahmed N., Chakrabarty A., Guengerich F. P., Chowdhury G. (2020). Protective role of glutathione against peroxynitrite-mediated DNA damage during acute inflammation. *Chemical Research in Toxicology*.

[17] Taylor E. W., Radding W. (2020). Understanding selenium and glutathione as antiviral factors in COVID-19: does the viral Mpro protease target host selenoproteins and glutathione synthesis?. *Frontiers in Nutrition*.

[18] Polonikov A. (2020). Endogenous deficiency of glutathione as the most likely cause of serious manifestations and death in COVID-19 patients. *ACS Infectious Diseases*.

[19] Khanfar A., Al Q. B. (2020). Could glutathione depletion be the Trojan horse of COVID-19 mortality?. *European Review for Medical and Pharmacological Sciences*.

[20] Guloyan V., Oganesian B., Baghdasaryan N. (2020). Glutathione supplementation as an adjunctive therapy in COVID-19. *Antioxidants (Basel)*.

[21] Yang Z., Shi J., He Z. (2020). Predictors for imaging progression on chest CT from coronavirus disease 2019 (COVID-19) patients. *Aging (Albany NY)*.

[22] Peng S., Chen J., Zhang W. (2021). The role of chest CT quantitative pulmonary inflammatory index in the evaluation of the course and treatment outcome of COVID-19 pneumonia. *Scientific Reports*.

[23] Huang Y., Tan C., Wu J. (2020). Impact of coronavirus disease 2019 on pulmonary function in early convalescence phase. *Respiratory Research*.

[24] Sun Y., Dong Y., Wang L. (2020). Characteristics and prognostic factors of disease severity in patients with COVID-19: the Beijing experience. *Journal of Autoimmunity*.

[25] Ivanov A. V., Alexandrin V. V., Paltsyn A. A. (2018). Metoprolol and nebivolol prevent the decline of the redox status of low-molecular-weight aminothiols in blood plasma of rats during acute cerebral ischemia. *Journal of Cardiovascular Pharmacology*.

[26] Witko-Sarsat V., Friedlander M., Capeillère-Blandin C. (1996). Advanced oxidation protein products as a novel marker of oxidative stress in uremia. *Kidney International*.

[27] Lei X., Du L., Yu W., Wang Y., Ma N., Qu B. (2021). GSTP1 as a novel target in radiation induced lung injury. *Journal of Translational Medicine*.

[28] Hughes M. M., McGettrick A. F., O'Neill L. A. J. (2017). Glutathione and glutathione transferase omega 1 as key posttranslational regulators in macrophages. *Microbiology Spectrum*.

[29] Ndrepepa G., Kastrati A. (2016). Gamma-glutamyl transferase and cardiovascular disease. *Annals of Translational Medicine*.

[30] Burns M., Rizvi S. H. M., Tsukahara Y. (2020). Role of glutaredoxin-1 and glutathionylation in cardiovascular diseases. *International Journal of Molecular Sciences*.

[31] Lepedda A. J., Formato M. (2020). Oxidative modifications in advanced atherosclerotic plaques: a focus on in situ protein sulfhydryl group oxidation. *Oxidative Medicine and Cellular Longevity*.

[32] Bartolini D., Giustarini D., Pietrella D., Rossi R., Galli F. (2020). Glutathione S-transferase P influences the Nrf2-dependent response of cellular thiols to seleno-compounds. *Cell Biology and Toxicology*.

[33] Pastore A., Piemonte F. (2013). Protein glutathionylation in cardiovascular diseases. *International Journal of Molecular Sciences*.

[34] Nonaka K., Kume N., Urata Y. (2007). Serum levels of S-glutathionylated proteins as a risk-marker for arteriosclerosis obliterans. *Circulation Journal*.

[35] Adachi T., Weisbrod R. M., Pimentel D. R. (2004). S-Glutathiolation by peroxynitrite activates SERCA during arterial relaxation by nitric oxide. *Nature Medicine*.

[36] Clavreul N., Adachi T., Pimental D. R., Ido Y., Schöneich C., Cohen R. A. (2006). S-Glutathiolation by peroxynitrite of p21ras at cysteine-118 mediates its direct activation and downstream signaling in endothelial cells. *The FASEB Journal*.

[37] Han J., Weisbrod R. M., Shao D. (2016). The redox mechanism for vascular barrier dysfunction associated with metabolic disorders: glutathionylation of Rac1 in endothelial cells. *Redox Biology*.

[38] Pincemail J., Cavalier E., Charlier C. (2021). Oxidative stress status in COVID-19 patients hospitalized in intensive care unit for severe pneumonia. A pilot study. *Antioxidants (Basel)*.

[39] Hati S., Bhattacharyya S. (2020). Impact of thiol-disulfide balance on the binding of Covid-19 spike protein with angiotensin-converting enzyme 2 receptor. *ACS Omega*.

[40] Chang R., Mamun A., Dominic A., le N. T. (2021). SARS-CoV-2 mediated endothelial dysfunction: the potential role of chronic oxidative stress. *Frontiers in Physiology*.

[41] Mak T. W., Grusdat M., Duncan G. S. (2017). Glutathione primes T cell metabolism for inflammation. *Immunity*.

[42] Hadzic T., Li L., Cheng N., Walsh S. A., Spitz D. R., Knudson C. M. (2005). The role of low molecular weight thiols in T lymphocyte proliferation and IL-2 secretion. *Journal of Immunology*.

[43] Franco R., Panayiotidis M. I., Cidlowski J. A. (2007). Glutathione Depletion Is Necessary for Apoptosis in Lymphoid Cells Independent of Reactive Oxygen Species Formation. *The Journal of Biological Chemistry*.

[44] Abraham E. C., Taylor J. F., Lang C. A. (1978). Influence of mouse age and erythrocyte age on glutathione metabolism. *The Biochemical Journal*.

[45] Van Lieshout E., Peters W. (1998). Age and gender dependent levels of glutathione and glutathione S-transferases in human lymphocytes. *Carcinogenesis*.

[46] Samiec P., Drews-Botsch C., Flagg E. (1998). Glutathione in human plasma: decline in association with aging, age-related macular degeneration, and diabetes. *Free Radical Biology & Medicine*.

[47] Jain S. K., Parsanathan R. (2020). Can vitamin D and L-cysteine co-supplementation reduce 25(OH)-vitamin D deficiency and the mortality associated with COVID-19 in African Americans?. *Journal of the American College of Nutrition*.

[48] Murai I. H., Fernandes A. L., Sales L. P. (2021). Effect of a single high dose of vitamin D3 on hospital length of stay in patients with moderate to severe COVID-19: a randomized clinical trial. *Journal of the American Medical Association*.

[49] Sims A. C., Tilton S. C., Menachery V. D. (2013). Release of severe acute respiratory syndrome coronavirus nuclear import block enhances host transcription in human lung cells. *Journal of Virology*.

[50] Parsanathan R., Jain S. K. (2019). Glutathione deficiency induces epigenetic alterations of vitamin D metabolism genes in the livers of high-fat diet-fed obese mice. *Scientific Reports*.

[51] Lu X., Ma Y., He J., Li Y., Zhu H., Yu X. (2019). N-Acetylcysteine for adults with acute respiratory distress syndrome: a meta-analysis of randomized controlled trials. *Hong Kong Journal of Emergency Medicine*.

[52] Zhang Y., Ding S., Li C., Wang Y., Chen Z., Wang Z. (2017). Effects of N-acetylcysteine treatment in acute respiratory distress syndrome: a meta-analysis. *Experimental and Therapeutic Medicine*.

[53] Rostami M., Mansouritorghabeh H. (2020). D-dimer level in COVID-19 infection: a systematic review. *Expert Review of Hematology*.

[54] Li Y., Zhao K., Wei H. (2020). Dynamic relationship between D-dimer and COVID-19 severity. *British Journal of Haematology*.

[55] Ibrahim H., Perl A., Smith D. (2020). Therapeutic blockade of inflammation in severe COVID-19 infection with intravenous N-acetylcysteine. *Clinical Immunology*.

[56] Horowitz R. I., Freeman P. R., Bruzzese J. (2020). Efficacy of glutathione therapy in relieving dyspnea associated with COVID-19 pneumonia: a report of 2 cases. *Respiratory Medicine Case Reports*.

[57] Lanini S., Montaldo C., Nicastri E. (2020). COVID-19 disease-temporal analyses of complete blood count parameters over course of illness, and relationship to patient demographics and management outcomes in survivors and non-survivors: a longitudinal descriptive cohort study. *PLoS One*.

[58] Koenig G., Seneff S. (2015). Gamma-glutamyltransferase: a predictive biomarker of cellular antioxidant inadequacy and disease risk. *Disease Markers*.

[59] Lee D. H., Jacobs D. R. (2005). Association between serum gamma-glutamyltransferase and C-reactive protein. *Atherosclerosis*.

